# Reduced polyfunctional T cells and increased cellular activation markers in adult allergy patients reporting adverse reactions to food

**DOI:** 10.1186/s12865-020-00373-w

**Published:** 2020-07-22

**Authors:** Friederike Sonnet, Ellen Namork, Eva Stylianou, Ingvild Gaare-Olstad, Kanutte Huse, Sandra Andorf, Siri Mjaaland, Hubert Dirven, Unni Nygaard

**Affiliations:** 1 Department of Toxicology and Risk Assessment, Norwegian Institute of Public Health, Lovisenberggata 8, Oslo, Norway; 2 Utrecht, the Netherlands; 3 Regional Unit for Asthma, Allergy and Hypersensitivity, Department of Pulmonary Diseases, Oslo University Hospital, Kirkeveien 166, Oslo, Norway; 4 Department of Cancer Immunology, Oslo University Hospital, Ullernchausseen 70, Oslo, Norway; 5 Sean N. Parker Center for Allergy and Asthma Research, Stanford University School of Medicine, 291 Campus Drive, Stanford, CA USA; 6 Department of Infectious Diseases Epidemiology and Modelling, Norwegian Institute of Public Health, Lovisenberggata 8, Oslo, Norway; 7 K.G. Jebsen Center for Influenza Vaccine Research Oslo, Kirkeveien 166, Oslo, Norway

**Keywords:** Food allergy, Adverse reactions to food, Specific IgE, Polyfunctional cells, CyTOF/mass cytometry, CITRUS algorithm

## Abstract

**Background:**

The underlying cellular mechanisms causing adverse reactions to food are complex and still not fully understood. Therefore, in this study we aimed to identify functional and/or phenotypical immune cell signatures characteristic for adult patients reporting adverse reactions to food.

By mass cytometry, we performed high-dimensional profiling of peripheral blood mononuclear cells (PBMC) from adult patients reporting adverse reactions to food and healthy controls. The patients were grouped according to sIgE-positive or sIgE-negative serology to common food and inhalant allergens. Two broad antibody panels were used, allowing determination of major immune cell populations in PBMC, as well as activation status, proliferation status, and cytokine expression patterns after PMA/ionomycin-stimulation on a single cell level.

**Results:**

By use of data-driven algorithms, several cell populations were identified showing significantly different marker expression between the groups.

Most striking was an impaired frequency and function of polyfunctional CD4+ and CD8+ T cells in patients reporting adverse reactions to food compared to the controls. Further, subpopulations of monocytes, T cells, and B cells had increased expression of functional markers such as CD371, CD69, CD25, CD28, and/or HLA-DR as well as decreased expression of CD23 in the patients. Most of the differing cell subpopulations were similarly altered in the two subgroups of patients.

**Conclusion:**

Our results suggest common immune cell features for both patient subgroups reporting adverse reactions to food, and provide a basis for further studies on mechanistic and diagnostic biomarker studies in food allergy.

## Background

Food allergy has emerged as a considerable public health concern, affecting up to 0.1–5.7% of children and adolescents under 18, and 0.1–3.2% of adults in westernized countries [1]. Food allergic reactions are either mediated by an immunological mechanism [2], involving allergen-specific Immunoglobulin E (sIgE), cell-mediated mechanisms in the absence of sIgE in serum or may show etiologies of both [3, 4]. The underlying cellular mechanisms causing adverse reactions to food in subjects with a suspected food allergy but where sIgE is not detected are largely unknown [5]. In Norway, about 50% of cases reported to The Norwegian Register of Adverse Reactions to Food did not have detectable sIgE to a standard panel of 12 food allergens in serum. Most of these cases (95%) were also negative for sIgE to inhalant allergens [6].

Diagnosing food allergy is highly challenging and complex. A careful assessment of the clinical history is currently the most important tool for the diagnosis of food allergy [7]. Although oral food challenges (especially the double-blind placebo-controlled food challenge) are the golden standard for an objective diagnosis of food allergy, food challenges are infrequently conducted outside the academic context as the procedure is resource-intensive, requires highly equipped specialists, and carries the risk of inducing a severe anaphylactic reaction [8, 9]. If an IgE-mediated food allergy is suspected, assessment of sIgE either in blood or via skin prick tests is recommended to identify the offending food. However, the relation between sIgE serum levels and adversity of the allergic responses varies, thus sIgE can occur in subjects without clinical food allergy symptoms and vice versa [10–12]. Also, other serum markers, such as IgG4 or cytokines, have been evaluated but were not validated as reliable diagnostic markers [13, 14].

Whereas a few cellular in vitro procedures have been considered to support diagnosis [4], there are still large knowledge gaps regarding cellular mechanisms.

Recent advances in cell cytometry, combining high-dimensional assessment of cellular phenotype and function with data-driven statistical algorithms [15, 16] allow for capturing the complexity of cellular immune mechanisms in a new scope. In this regard, our objective in this explorative study was to identify phenotypical and/or functional immune cell signatures characteristic for patients reporting adverse reactions to food. The overarching goal was to obtain new insight into the cellular mechanisms of food allergy, contributing to a more accurate clinical diagnosis.

By the use of mass cytometry/CyTOF (cytometry by time of flight), we performed comprehensive profiling of peripheral blood mononuclear cells (PBMC) from adult patients reporting adverse reactions to food and healthy controls. The patients were grouped according to sIgE-positive or sIgE-negative serology to common food and inhalant allergens. Using a combination of manual gating strategies and data-driven approaches [15], immune cell profiles and functional cell subpopulations differing between the groups of participants were identified.

## Results

### Serum analysis

In all collected blood samples, sIgE to 12 common food allergens (milk, egg, wheat, pea, soy, peanut, fenugreek, hazelnut, celery, cod, salmon and shrimp) were analyzed, as well as sIgE to rx6 (pollens from birch, timothy, mugwort, and mold (*cladosporium* and *alternaria*)) and rx7 (mite (*D. pteronyssinus*), cat dander, horse, dog, and rabbit epithelium) inhalant allergens (summarized in Table 1, serum levels reported in S. Table I). The control subjects (*n* = 8) had no detectable sIgE to any of the allergens tested. The patients reporting adverse reactions to food were assigned to two groups based on the presence or absence of any sIgE to these food and inhalant allergens, hereafter called the IgEpos (*n* = 11) and IgEneg (*n* = 9) allergy groups. This grouping was confirmed by the presence or absence of sIgE also at the time of reporting to The Food Allergy Register (data not shown).

**Table 1:** Characteristics of the study subjects

	Age	Sex	Asthma	Symptoms^a^					sIgE to food allergens ^c^	sIgE to pollens and mold^d^	sIgE to mite and animals’ dander/ epithelium^e^
	(yr)			A	B	C	D	E		(rx6)	(rx7)
*IgEneg donors*											
N1	31	F	No	2	1	1			neg	neg	neg
N2	20	M	No	1	2	1			neg	neg	neg
N3	63	M	No		2			1	neg	neg	neg
N4	68	F	Yes		1		2		neg	neg	neg
N5	54	F	No	1		2	1		neg	neg	neg
N6	42	F	No	2	3				neg	neg	neg
N7	68	F	Yes	1	2			1	neg	neg	neg
N8	66	F		missing					neg	neg	neg
N9	61	M	No	1	2			1	neg	neg	neg
Group median	**59**										
*IgEpos donors*											
P1	36	F	Yes			3			haz	pos (birch/tim)	pos (cat/dog)
P2	45	F	No		1	2			haz	pos (birch/tim/mugw)	pos (cat/dog/horse)
P3	44	F	Yes	3	2	2		2	pea, peanut, soy, haz, wheat	pos (birch/tim/mugw)	neg
P4	43	F	Yes	2	1	2			neg	pos (tim/mugw)	pos (dog)
P5	43	F	No	2		2			pea, peanut, soy, haz, milk	pos (birch)	pos (cat)
P6	37	F	No	2					chicken	neg	neg
P7	31	F	No		1	1			haz	pos (birch/tim/mugw)	pos (cat/dog/horse)
P8	38	F	Yes		3		2	3	peanut, haz, milk, wheat, fenu, chili, parsley	pos (birch/tim/mugw)	pos (cat/dog/horse)
P9	62	M	No	2					shrimp	neg	pos (mite)
P10	32	F	No	2	2	2			peanut, haz, celery	pos (birch/tim)	pos (cat, dog, horse)
P11	24	M	Yes				3		pea, peanut, soy, haz, wheat, fenu	pos (birch/tim)	pos (cat, dog, horse)
Group median	**43**										
*Healthy donors*											
C1	57	F	No						neg	neg	neg
C2	46	F	No						neg	neg	neg
C3	47	M	No						neg	neg	neg
C4	58	M	No						neg	neg	neg
C5	40	F	No						neg	neg	neg
C6	40	F	No						neg	neg	neg
C7	28	M	No						neg	neg	neg
C8	41	F	No						neg	neg	neg
Group median	**42**										

Abbreviations: *F* female, *M* male, *neg* negative, *pos* positive, *n.r*. not relevant, *tim* timothy, *mugw* mugwort, *haz* hazelnut, *fenu* fenugreek

^a^Symptoms as reported to the food allergy register at time of the adverse reaction. A: skin, B: gastrointestinal tract, C: respiratory tract, D: cardiovascular system, E: neurological system; severity of symptoms: 1 = mild, 2 = moderate, 3 = severe

^b^Self reported, suspected offending food (reported to the food allergy register)

^c^Positive sIgE (> 0.35 kU/L in serum, analyzed by ImmunoCAP) to i) any of the 12 allergens in the standard panel (milk, egg, wheat, pea, soy, peanut, fenugreek, hazelnut, celery, cod, salmon and shrimp, as well as birch and timothy), ii) other allergens based on reported suspected offending food or iii) any allergen positive in the dot blot matrix. Negative sIgE denotes individuals without any detectable sIgE to the standard panel or the dot blot matrix. IgE levels in kU/L are given in supplementary Table 1

^d^rx6 comprises a mix of allergens from birch, timothy, mugwort pollens or mold (cladosporium and alternaria). If positive for sIgE to rx6, also the single allergens were analyzed by ImmunoCAP, and allergens with positive sIgE given in paranthesis. IgE levels in kU/L are given in supplementary Table 1

^e^rx7 comprises a mix of allergens from mite (*D. pteronyssinus*), dander from cat, horse, dog or rabbit epithelium. If positive for sIgE to rx7, also the single allergens were analyzed by ImmunoCAP, and allergens with positive sIgE given in parentheses. IgE levels in kU/L are given in supplementary Table 1

*medication, alcohol and exercise are reported and may aggravate food allergic responses [17]

Participant characteristics are summarized in Table 1. The subjects in the control group, the IgEpos, and the IgEneg groups, were of both genders and ranging in age between 28 and 58, 20–68, and 24–62 years, respectively. Among the patients reporting adverse reactions to food with available clinical data at the time of reporting to the Food Allergy Register, all presented mild to severe symptoms affecting mostly skin, respiratory tract, and/or gastrointestinal tract (Table 1).

Total IgE and IgG4 in the participant’s sera, as well as the IgG4/IgE ratio, did not significantly differ between the groups (Suppl. Table I). There were also no significant differences between the groups for TNF-α, IL-6, IL-8 and MCP-1 in serum (S. Table I), while IL-1b serum levels were below the detection limit for most of the samples (data not shown).

### Immunophenotyping of unstimulated cells – using antibody panel 1

**Fig. 1:**
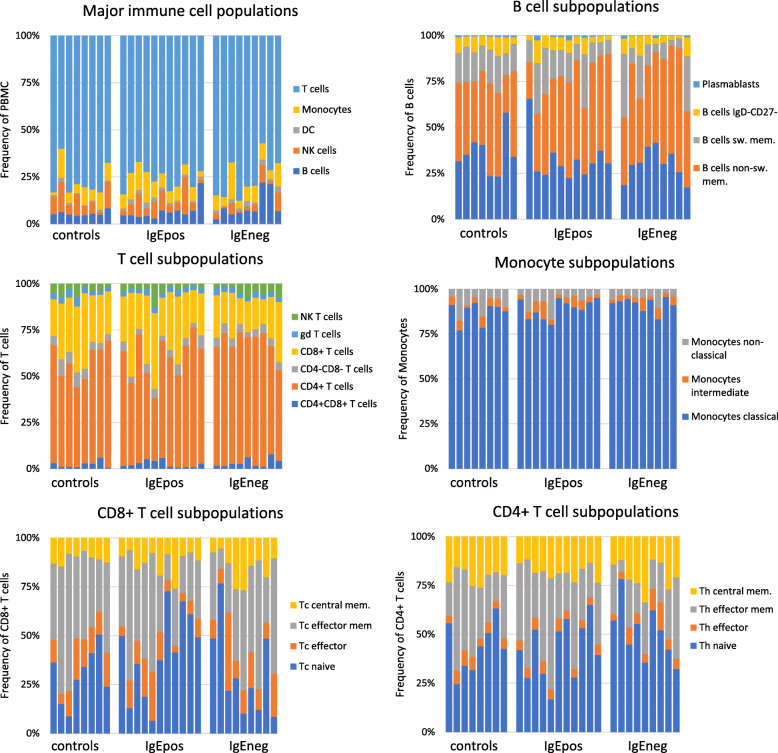
Individual frequencies of immune cell subpopulations obtained by traditional manual gating of populations according to Table 3 in healthy controls, IgEpos food allergy subjects, and IgEneg food allergy subjects

Manual biaxial gating (Table 3) assessed the frequencies of traditionally defined immune cell lineages (T cells, B cells, monocytes, NK cells, and DC). No statistically significant differences were detected between the control group and the two allergy groups, nor between the IgEpos and IgEneg group in any of these cell populations (Fig. 1).

In the CITRUS analyses, where cells are clustered based on their overall marker similarities, the abundances of cells in the cell clusters generated by clustering on all 33 surface markers (25 phenotyping and 8 functional/activation markers) or by clustering on the 25 phenotyping markers were not statistically significantly different between the three groups.

**Fig. 2:**
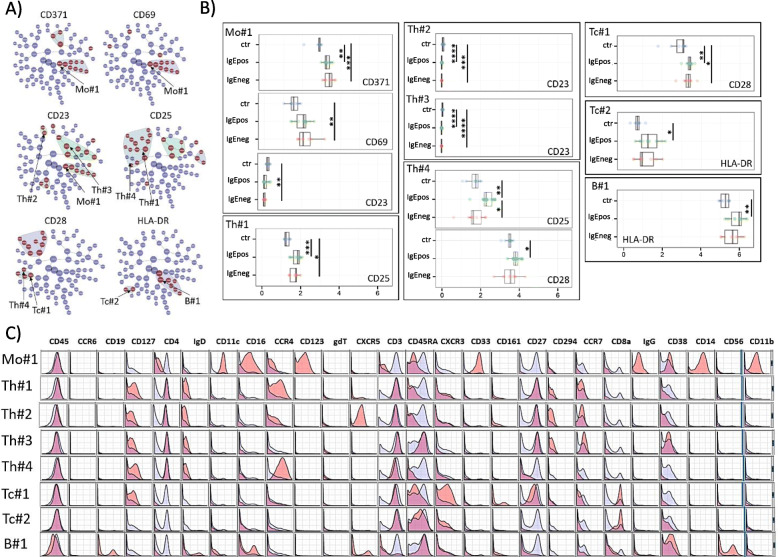
CITRUS analyses for the unstimulated cells, clustering on 25 phenotyping markers and comparing median expression of the markers CD371, CD23, CD25, CD28, and HLA-DR between the three groups. **a** CITRUS trees in which each node denotes different cell clusters. The red nodes illustrate cell populations were the median marker intensities of the respective functional marker differed statistically significantly between the three groups as determined by SAM analyses, FDR 0.05. The parent clusters (the “highest in the hierarchy” significant node) are named based on marker expression shown in C. **b** Median marker intensities for the various functional markers, shown as box plots (expressing interquartile range (IQR) and median values) and values for each individual within the groups of healthy controls, IgEpos, and IgEneg food allergic subjects, for each parent clusters identified in A. Differences between groups are shown as lines, * *P* ≤ 0.05, ** *P* ≤ 0.01, *** *P* ≤ 0.001, and **** *P* ≤ 0.0001 (Kruskal-Wallis and Dunn’s multiple comparisons test). **c** CITRUS histograms for the parent clusters identified in A. For each surface marker, the histogram shows the marker expression on cells in the specific cluster (red) against the marker expression on all other cells (blue) – overlapping of histograms will result in purple areas

However, when assessing median marker intensities of the functional/activation markers, indicative for the quantitative marker expression per cell, several differences between the groups were observed. After clustering on the 25 phenotyping markers, the expression of CD371, CD69, CD23, CD25, CD28, and HLA-DR differed for nine different parent clusters (parent cluster denoting the statistically significantly stratifying cluster being highest in hierarchy) and several generations of child clusters (Fig. 2a).

Cells in the eight parent clusters were identified as subpopulations of monocytes (Mo#1), Th cells (Th#1, Th#2, Th#3, Th#4), Tc cells (Tc#1, Tc#2), and B cells (B#1). The phenotypes of these subpopulations are presented as marker histograms in Fig. 2c.

Monocytes in the Mo#1 cluster were CD28^low^ CD69^high^ HLA-DR^high^ CD371^high^ CD23^low^ CD123^high^. Compared to the control group, this monocyte subpopulation had a statistically significantly increased expression of CD371 in both allergy groups, a statistically significantly increased expression of CD69 in the IgEneg group, and a decreased, although overall low, expression of CD23, reaching statistical significance only in the IgEneg group (Fig. 2b). In support, up to five generations of child clusters had similar patterns of statistically significant differences with regard to the expression of CD371 and CD69, and CD23 (Fig. 2a, S. Table II).

Th cells in cluster Th#1 were identified as being predominantly of the naïve and central memory type with a high expression of the Th2 cell marker CRTH2, being CD28^high^ CD69^low^ and CD25^+^. Compared to the controls, these cells had a statistically significantly increased expression of CD25 in both allergy groups, which tended to be stronger in the IgEpos group (Fig. 2b). In support, up to four generations of child clusters had a similar pattern regarding CD25 expression (Fig. 2a, S. Table II).

Like the monocytes above, Th cells in the clusters Th#2 and Th#3 had a statistically significantly decreased expression of CD23 in both allergy groups compared to the controls, supported by one to three generations of child clusters, although the expression of CD23 was in general very low in these clusters (Fig. 2a, b). Both Th#2 and Th#3 cells were expressing CRTH2 and being CD28^high^ CD69^low^ and CD25^low/+^. Cells in the Th#2 cluster were predominantly of the naïve and central memory type, while cells in the Th#3 cluster were of the naïve type (Fig. 2c, S. Table II).

Th cells in the Th#4 cluster were predominantly of the effector memory and effector type being CD28^high^ CD69^low^ CD25^+^ CD134^low^ CD163^low^ CD371^low^. Like the Th#1 cells, these Th#4 cells had an increased expression of CD25 in the IgEpos allergy group, being statistically significantly higher than the controls and in the IgEneg allergy group. The IgEpos group additionally had a statistically significantly increased expression of CD28 compared to the controls (Fig. 2a, b).

Tc cells in cluster Tc#1 were predominantly of the naïve and effector type being CD28^high^ CD69^low^ HLA-DR^+^ (Fig. 2a, S. Table II). In both allergy groups, the expression of CD28 was statistically significantly increased compared to the control group, supported by two generations of child clusters (Fig. 2b).

Tc cells in theTc#2 and B cells in the B#1 clusters both had an increased expression of HLA-DR in the allergy groups compared to the controls, reaching statistical significance only in the IgEpos group (Fig. 2b). Tc#2 cells were identified as being predominantly of the naïve and effector type being CD28^+^ CD69^+^ HLA-DR^+^ while the B#1 cells were predominantly of the naïve type being CD28^low^ CD69^+^ CD25^low^ HLA-DR^high^. Tc#2 cell population had one child cluster and the B#1 cell cluster had up to four generations of child clusters with the same patterns for HLA-DR expression in the three groups (Fig. 2a, S. Table II).

### Functional assessment of stimulated cells – using antibody panel 2

To assess functional differences between cells from the three groups of participants, intracellular cytokine expression and proliferation (Ki67) were assessed in addition to the main surface phenotyping markers, after cell stimulation with PMA and ionomycin.

**Fig. 3:**
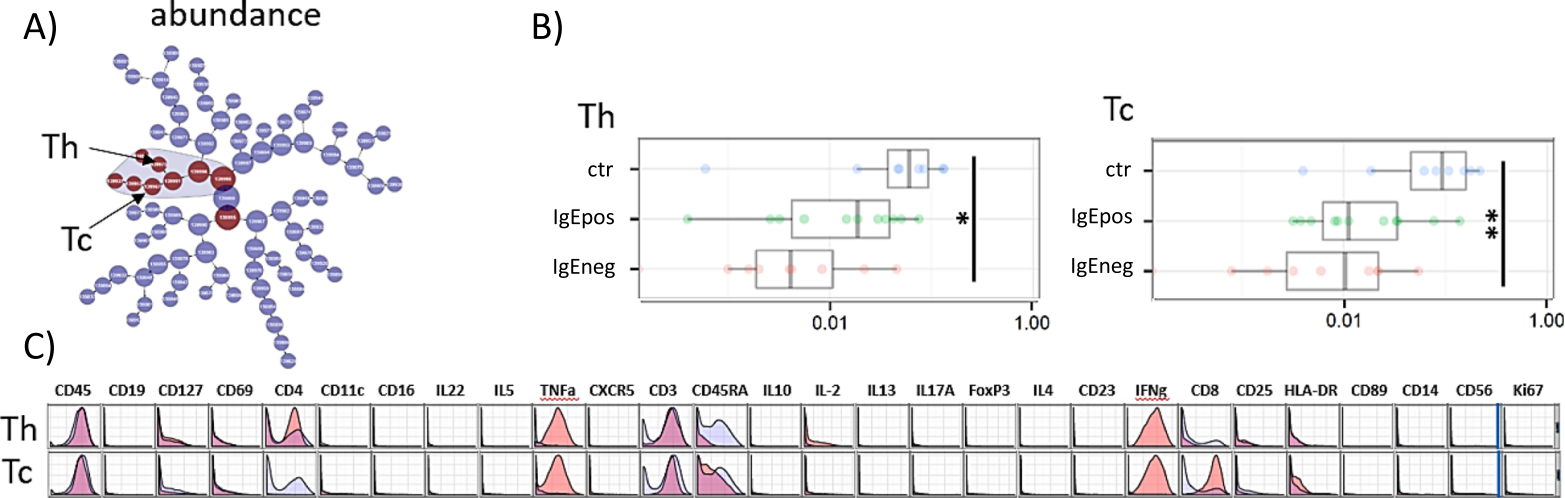
CITRUS analyses for the stimulated cells, clustering on all 28 surface and intracellular markers and comparing cell abundance in the three groups. **a** CITRUS tree in which each node denotes different cell clusters. The red nodes illustrate cell populations were the cell abundance differed statistically significantly between the three groups as determined by SAM analyses, FDR 0.05. **b** Cell abundance as proportion of all cells, shown as box plots (expressing IQR and median values) and values for each individual within the groups of healthy controls, IgEpos, and IgEneg food allergic subjects, for the clusters identified in A. Differences between groups are shown as lines, * *P ≤* 0.05, ** *P ≤* 0.01, *** *P ≤* 0.001, and **** *P ≤* 0.0001 (Kruskal-Wallis and Dunn’s multiple comparisons test). **c** CITRUS histograms for the clusters identified in A. For each surface marker, the histogram shows the marker expression on cells in the specific cluster (red) against the marker expression on all other cells (blue) – overlapping of histograms will result in purple areas

When clustering on all 28 markers, statistically significant differences between the groups were identified in a branch of six parent/child cell clusters all being CD3^+^ T cells co-expressing high levels of TNF-α and IFN-γ (Fig. 3a). The abundance of these cells was reduced in both allergy groups compared to the control group, although reaching statistical significance only in the IgEneg group (Fig. 3b, S. Table II). The parent cell cluster branched into two child clusters characterized as Th cells predominantly of the effector and effector memory type and Tc cells predominantly of the effector and to a lesser extent of the naïve type.

The Th cells population contained further subpopulations, also expressing IL-2 and/or CD25, whereas the last generation child cluster of the Tc cells contained subpopulations also expressing IL-2 and/or HLA-DR. The phenotype of the Th and Tc parent clusters is presented as marker histograms in Fig. 3c.

**Fig. 4:**
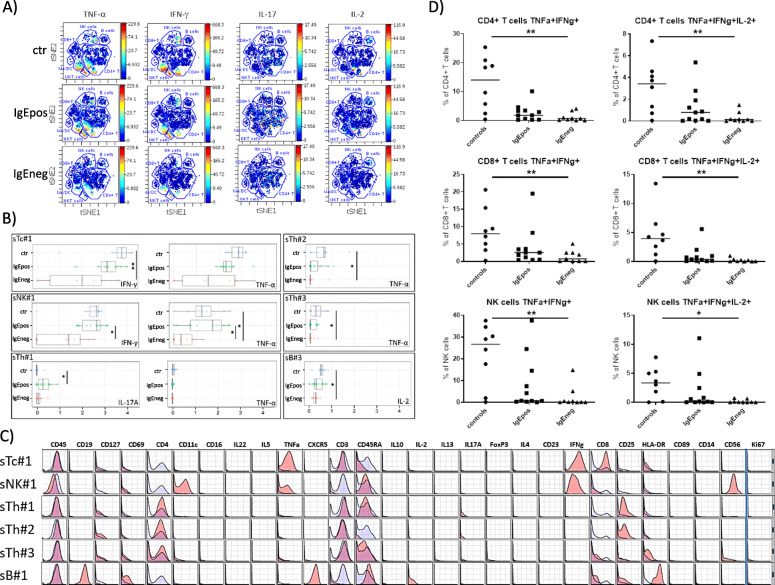
CITRUS analyses for the stimulated cells, clustering on all 28 surface and intracellular markers and comparing median expression of the markers TNF-α, IFN-γ, IL-17A, and IL-2 between the three groups. **a** viSNE maps of PBMC of one representative subject of the control, IgEpos, and IgEneg allergy group, cells are colored by TNF-α, IFN-γ, IL-17A, and IL-2 expression respectively. **b** Median marker intensities for the various functional markers, shown as box plots and values for each individual within the groups of healthy controls, IgEpos, and IgEneg food allergic subjects, for each parent clusters identified in A. Differences between groups are shown as lines, * *P ≤* 0.05, ** *P ≤* 0.01, *** *P ≤* 0.001, and **** *P ≤* 0.0001 (Kruskal-Wallis and Dunn’s multiple comparisons test). **c** CITRUS histograms for the major parent clusters. For each surface marker, the histogram shows the marker expression on cells in the specific cluster (red) against the marker expression on all other cells (blue) – overlapping of histograms will result in purple areas. **d** Scatter plots of frequencies of polyfunctional CD4^+^ Th cells, CD8^+^ Tc cells, and NK cells simultaneously expressing TNF-α^+^ IFN-γ^+^ (left panel) and TNF-α^+^ IFN-γ^+^ IL-2^+^ (right panel) in the three groups of participants: controls, IgEpos food allergy patients, and IgEneg food allergy patients. Each dot represents a participant while the lines represent the group median. * denotes *P ≤* 0.05 and ** denotes *P ≤* 0.01 (Kruskal-Wallis and Dunn’s multiple comparisons test).

We further investigated group differences in the median marker intensities of CD69, Ki67, and the cytokines IL-2, IL-4, IL-5, IL-10, IL-13, IL-17A, IL-22, IFN-γ, and TNF-α. CITRUS identified group differences in the expression of TNF-α, IFN-γ, IL-17A, and IL-2. Most striking was the decreased (co) expression of TNF-α in numerous cell clusters in the two allergy groups compared to the controls. The differences in the median marker intensities are visualized for one representative subject of each group in viSNE maps Fig. 4a. The phenotype of the cells in these clusters is presented as histograms in Fig. 4c.

Cell clusters showing a statistically significantly (in SAM) simultaneous reduction in the expression of TNF-α and IFN-γ were identified as subpopulations of Tc cells (stimulated (s)Tc#1) and NK cells (sNK#1). In the Tc#1 subpopulation, only the reduced expression of IFN-γ in the IgEneg group was reaching statistical significance in the pairwise Kruskal-Wallis test compared to the controls. In the NK#1 cells, the expression of TNF-α was statistically significantly decreased in the IgEneg group compared to the controls and the IgEpos group, whereby the expression of IFN-γ was statistically significantly decreased only in the IgEneg group compared to the IgEpos group (Fig. 4b,c, S. Table II). The sNK#1 cell cluster had up to three generations of child clusters and the sTc#1 cell cluster had one child cluster with the same significance pattern regarding TNF-α and IFN-γ expression (data not shown).

A subpopulation identified as Th cells (sTh#1) was significantly differing between allergy groups and the control group in the co-expression of TNF-α and IL-17A. These Th cells were characterized as being predominantly of the naïve type and to a lesser extent of the effector type and showed a tendency of a reduced expression of TNF-α in both allergy groups, although not reaching statistical significance, and an increased expression of IL-17A in the allergy groups, statistically significant only in the IgEpos group (Fig. 4b,c, S. Table II). Up to two generations of child clusters had the same pattern regarding TNF-α and IL-17A expression (data not shown).

Two other subpopulations of Th cells (sTh#2, sTh#3) differed in the expression of TNF-α only (Fig. 4b). In both clusters, TNF-α was expressed in low levels, but was statistically significantly decreased in the IgEneg group, whereby the IgEpos group had the same tendency. (Fig. 4b, c, S. Table II). Cells in the sTh#3 cluster were characterized as being predominantly of the naive and effector type (Fig. 4b, c, S. Table II). In support, up to five generations of child clusters of the sTh#2 cluster and two generations of child clusters of the sTh#3 clusters had a similar pattern regarding TNF-α expression (data not shown).

Lastly, one cluster differing in the expression of IL-2 was identified as a population of B cells (sB#1). B cells in that cluster had a decreased expression of IL-2 in the allergy groups, reaching statistical significance only in the IgEneg group (Fig. 4b, c, S. Table II).

No group differences in expression of the other cytokines were observed.

After manual gating of CD4+ T, CD8+ T and NK cells according to S. Figure IIA, there was a reduction of TNF-α + IFN-γ+, IL-2+ cells in both allergy groups, reaching statistical significance only in the IgEneg allergy group (Fig. 4d). In Th cells, the group median percentage of TNF-α + IFN-γ + IL-2+ cells was 3.42% in the control group, 0.80% in the IgEpos group, and 0.14% in the IgEneg group, while the group median percentage of TNF-α + IFN-γ + cells was 14, 1.85, and 0.71%, respectively. In Tc cells, the group median percentage of TNF-α + IFN-γ + IL-2+ cells was 3.94% in the control group, 0.39% in the IgEpos group, and 0.05% in the IgEneg group, while the group median percentage of TNF-α + IFN-γ + cells was 7.96, 2.56, and 0.77%, respectively. In NK cells, the group median percentage of TNF-α + IFN-γ + IL-2+ cells was 3.34% in the control group, 0.15% in the IgEpos group, and 0.04% in the IgEneg group, while the median percentage of TNF-α + IFN-γ + cells was 26.66, 0.80, and 0.15%, respectively (Fig. 4d). Cell percentages and median marker intensities for each of the cytokines individually are reported in S. Fig. II.

## Discussion

By the use of broad antibody panels and data-driven analyses, we identified several cell populations where the combination of marker expression was significantly different between individuals with adverse reactions to food (both sIgE-positive and sIgE-negative to common food and inhalant allergens), and healthy controls. While no differences were seen for more traditionally targeted serum markers (antibodies and proteins/cytokines), our results illustrate the great potential of high-dimensional analyses on single-cell level, such as mass cytometry, for the identification of new diagnostic biomarkers and/or mechanistic knowledge.

Whereas cell population phenotypic markers alone did not differ between the groups, several functional markers, such as the expression of activation markers in unstimulated cells, or (co) expression of cytokines in PMA/ionomycin-stimulated cells did differ. Interestingly, most of the cell features were similar in the patients reporting adverse reactions to food regardless of the presence of sIgE in serum.

Most IgEpos patients, except for P11 and P9, had sIgE to both food and inhalant allergens. In spite of the suspicion of cross-allergies, the results suggest that the cellular signature and several immune cell mechanisms may be common for these two groups of patients. Furthermore, it indicates that the group of sIgE-neg patients indeed were food allergic patients according to the nomenclature in Johansson et al. (2001), defined by responses mediated by an immunological mechanism. Although we cannot exclude that the food allergy patients in the IgE-neg group have sIgE in serum not detected by our ImmunoCap panel and food extract dot blot matrix, our results indicate that cellular mechanisms may be important and that cellular features may serve as helpful diagnostic markers of food allergy. Our results also underline the large knowledge gap of the underlying cellular mechanisms of hypersensitivity reactions in general.

The most striking observation was an apparent impairment of polyfunctionality of both Th (CD4^+^) and Tc (CD8^+^) cells in the food allergic individuals compared to healthy controls, although only reaching statistical significance in the IgEneg group. This result was consistent, with both lower abundance of TNF-α and IFN-γ producing Th and Tc cell subpopulations as well as lower expression of these cytokines per cell. Some of the subpopulations of these polyfunctional Th and Tc cells also co-expressed IL-2.

Likewise, TNF-α and IFN-γ co-expression was lower in NK cells. The effects on polyfunctional cells were supported by a similar pattern in several groups of parent-child clusters. In agreement, reduction in TNF-α and IFN-γ individually was observed even after manual gating on NK cells, CD4^+^ and CD8^+^ T cells. Further, we can exclude the possibility that the reduction in the two cytokines was not due to methodological errors such as lack of antibody staining or other systematic errors since other cell clusters with high expression of TNF-α and/or IFN-γ were not reduced in the allergy groups.

Polyfunctional cells are cells simultaneously expressing two or more immune mediators (cytokines/chemokines) [18], and have been described for CD4^+^ and CD8^+^ T cells, NK cells and monocytes [19, 20]. Polyfunctional cells have been shown to provide a more effective immune response to various pathogens such as human immunodeficiency virus (HIV) [21], *Leishmania major* [22], and *Mycobacterium tuberculosis* infections [23], than cells that produce only single cytokines, and reflect functional efficiency in vaccination [24]. Polyfunctional T cells have also been shown to play a role in certain autoimmune diseases [25]. Functional consequences of lower levels of polyfunctional T cells in food allergy may, therefore, be hypothesized. On the other hand, the lower abundance and TNF-α/IFN-γ cytokine response to PMA/ionomycin could also be a result of cell exhaustion in the observed Th, Tc, and NK cell populations [26–28] and/or Th2-skewing of T cell responses in the two allergy groups, as would be expected in particular for the IgEpos group [29]. The observation can depend on the choice of PMA/ionomycin as the stimulant since the stimulus strongly influences the immune signature [30]. Nevertheless, our results indicate that certain cell populations from the two allergy groups respond with altered ability for combined cytokine production compared to the control group in the present setup. This points to polyfunctional cells as a potential diagnostic biomarker for food allergy and deserves focus in future studies**.**

Both TNF-α and IFN-γ have previously been reported to be relevant for food allergic responses [23]. In agreement with our current findings, Osterlund et al. have reported decreased frequencies of IFN-γ expressing CD4^+^ T cells [31] and decreased production of TNF-α in culture supernatants of PBMC from children with cow’s milk allergy [32].

CITRUS did not detect expression of the Th2 cell cytokines IL-5, IL-10, or IL-13, cytokines that are strongly associated with food allergy [33]. The reason for this could be the type of stimuli, as described above, or the low frequencies of allergen-specific cells taking the limited amount of acquired cells into consideration [34].

In unstimulated cells, the cell count within each subpopulation did not differ significantly between the groups, neither the percentage of the conventional cell populations identified by manual gating nor in populations clustered based on all markers or only the phenotypic markers (by unsupervised clustering in CITRUS). However, for food allergic individuals, the expression of the activation/functional markers CD371, CD69, CD28, HLA-DR, and CD25 per cell was higher and CD23 was lower in several parent-child groups of cell subpopulations. The effects were observed in both allergy groups, and several of the activation markers were altered in the same cells. For instance, monocyte subpopulations showed higher levels of CD371 (inhibitory receptor [35]) and CD69 (early activation marker [36]) in both allergy groups and with simultaneously decreased CD23 (low affinity IgE receptor [17]) expression only in the IgEneg allergy group. Taken together with recent literature suggesting that monocytes might have a pivotal role in some non-IgE-mediated disorders [37], our observations suggest that the activation status of monocytes could be of interest for further studies of diagnostic markers and mechanisms of food allergy. Furthermore, also the higher expression of HLA-DR and CD28 are biologically relevant since they are involved in the crosstalk between antigen-presenting cells (e.g. B cells/DCs) and T cells during activation and crucial for the maintenance of immune homeostasis [38, 39]. Moreover, increased CD25 expression (high-affinity heterotrimeric IL-2 receptor [40]) might counterbalance the decreased expression of IL-2. Our data indicate that the expression of markers indicative of activation state and/or function are more potent in reflecting disease-dependent characteristics/features than cell population frequencies.

We exploited the power of unsupervised cluster analyses to identify subpopulations of cells with combinations of phenotypic and functional markers that were overlooked in a manual gating approach. While the choice of clustering algorithms and statistical analyses will to a certain degree influence the outcomes, our main findings were confirmed in different ways, strengthening the conclusions: i) by running all clustering analyses at least twice with similar results ii) in most cases, the results obtained by the SAM method for identifying cell clusters differing between the groups (with corrections for multiple comparisons) were confirmed by the PAMR results (data not shown), iii) the application of the relatively conservative Kruskal-Wallis test for pairwise comparisons between the three groups led to a reduction in significant cell populations reported.

The antibody panels were designed to cover a broad spectrum of different immune cells, but still only included a selection of markers to study activation, maturation, and proliferation status of the immune cells. In future studies, the use of whole blood should be considered, to assess neutrophils, basophils, and especially eosinophils, which are strongly associated with several disorders not associated with sIgE [5]. As suggested by Goswami et al. (2017), food-antigen specific cells with a pathogenic phenotype in allergic patients with an sIgE-negative serology might not be found in circulation, but be mainly localized to the gastrointestinal tract [37]. However, in the search for diagnostic markers, blood is the preferred matrix.

## Conclusion

In conclusion, we identified reduced polyfunctionality of Th, Tc and, NK cell subpopulations, as well as increased (co) expression of activation/functional surface markers in monocytes, but also T and B cell, subpopulations, in food allergic patients compared to controls. Most often, these changes were similar in patients with positive or negative sIgE serology to common food and inhalant allergens. Despite the small group sizes and the heterogeneity in the patients reporting adverse reactions to food, the consistency of the present observations provides directions for further studies on mechanistic and diagnostic biomarker searches for food allergy.

## Methods

### Study population

Adult patients (> 18 years) of both genders in the Greater Oslo Region were selected (*n* = 44), among doctors’ reports on patients with adverse reactions to foods sent to The Norwegian Register of Adverse Reactions to Food at the Norwegian Institute of Public Health, and invited to participate in the project. Reports had been received between 2000 and 2015. Routinely, all cases reported to the register were analyzed for sIgE to a standard panel of 12 food allergens and 2 inhalant allergens by ImmunoCAP [Thermo Fisher Scientific/Phadia, Uppsala, Sweden], as well as in an in-house dot blot matrix of 170 food extracts [6]. Positive sIgE responses in the non-validated dot blot matrix were subsequently quantified by ImmunoCAP for the corresponding allergen for verification.

Twenty patients accepted the invitation to participate in the present study, and donated blood at the Oslo University Hospital in 2016. At the time of reporting to the register, eleven of these study participants had positive sIgE to specific foods, while nine subjects neither had detectable sIgE in serum to the standard allergen panel nor to any foods in the matrix (data not shown). Eight healthy subjects of both genders were recruited at the Norwegian Institute of Public Health and assigned to the control (ctr) group. These subjects had no self-perceived or diagnosed asthma, food or inhalant allergies, or atopic eczema. The study was approved by the Norwegian Regional Committee for Medical and Health Research Ethics (2015/2070). Written, informed consent was obtained from all participants and all procedures were performed in accordance with approved guidelines.

### Blood samples and preparation of PBMC

The preparation of PBMC by density gradient centrifugation using Ficoll-Paque PLUS is described in detail in the supplementary information.

### Serum analysis

#### Measurements of total IgE and total IgG4 by ELISA

Total IgE and IgG4 in all participant’s sera were analyzed using human Platinum ELISAs, Ready-to-use Sandwich with 96-well strip plates [eBioscience, ThermoFisher Scientific] according to the manufacturers’ instructions. Absorbance of the ELISA reaction product was measured with a microplate reader [ELx808 absorbance reader, BioTek] at 450 nm, and the concentrations were determined from a standard curve included on each plate.

#### Specific IgE measurements by ImmunoCAP®

In all participants, serum concentrations of sIgE to the panel of 12 common food allergens were analyzed by standardized ImmunoCAP technology, as described previously [41]. The food allergens included were milk, egg, wheat, pea, soy, peanut, fenugreek, hazelnut, celery, cod, salmon, and shrimp. In addition, the screening tests for sIgE to the inhalant allergens rx6 (pollens from birch, timothy, mugwort, and mold (cladosporium and alternaria)) and rx7 (mite (*D. pteronyssinus*), cat dander, horse, dog, and rabbit epithelium) were performed for all participants, giving “positive” or “negative” as outcome based on a cutoff at 0.35 kU/L. When positive in these screening tests, the serum concentrations of sIgE to the individual allergens were also assessed by ImmunoCAP technology.

#### Measurements of serum cytokines by Luminex technology

Serum concentrations of the cytokines interleukin (IL)-6, IL-8, IL-1β and TNF-α and Monocyte Chemoattractant Protein-1 (MCP-1) were determined by Luminex [Bio-plex™ 200 Suspension Array System, with software Manager, version 6.1, Validation kit and Calibration kit, BioRad] using Human Adipokine Magnetic Bead Panel 2, 96 well plate assay, according to the manufacturer’s recommendations using undiluted serum [Milliplex® Map Kit, EMD Millipore Corporation].

### Mass Cytometry

Detailed information about the antibodies and reagents, thawing of PBMC, sample preparation for mass cytometry, acquisition, and data analysis id provided in the supplementary information.

The list of antibodies used is presented in Table 2, while in Table 3 cell subpopulations and the markers used for their identification are presented.

**Table 2:** Antibody specificity (target), metal tag and panel information sorted by metal tag. *denotes antibodies that were self-conjugated, ** seven stimulated samples did not contain this antibody, therefore, CCR7 was excluded from unsupervised analysis but used to determine T cell subpopulations in remaining 21 samples

	Isotope	Target
Panel 1 (32 surface antibodies)		
surface	089Y	CD45
surface	141Pr	CD196 (CCR6)
surface	142Nd	CD19
surface	143Nd	CD127 (IL-7Ra)
surface	144Nd	CD69
surface	145Nd	CD4
surface	146Nd	IgD
surface	147Sm	CD11c
surface	148Nd	CD16
surface	149Sm	CD194 (CCR4)
surface	151Eu	CD123 (IL-3R)
surface	152Sm	ydTcell receptor
surface	153Eu	CD185 (CXCR5)
surface	154Sm	CD3
surface	155Gd	CD45RA
surface	156Gd	CD183 (CXCR3)
surface	158Gd	CD33
surface	159 Tb	CD161
surface	160Gd	CD28
surface	162Dy	CD27
surface	163Dy	CD294 (CRTH2)
surface	164Dy	CD23
surface	167Er	CD197 (CCR7)
surface	168Er	CD8a
surface	169Tm	CD25 (IL-2R)
surface	170Er	HLA-DR
surface	172Yb	CD38
surface	173Yb	CD371 (CLEC12A)
surface	174Yb	*IgG
surface	175Lu	CD14
surface	176Yb	CD56 (NCAM)
surface	209Bi	CD11b (Mac-1)
intracellular	191Ir	Cell-ID intercalator
intracellular	193Ir	Cell-ID intercalator
Live/dead	194Pt	Cisplatin – live/dead
**Panel 2** (18 surface and 11 intracellular antibodies)		
surface	089Y	CD45
surface	142Nd	CD19
surface	143Nd	CD127 (IL-7Ra)
surface	144Nd	CD69
surface	145Nd	CD4
surface	147Sm	CD11c
surface	148Nd	CD16
intracellular	150Nd	IL-22
intracellular	151Eu	IL-5
intracellular	152Sm	TNFa
surface	153Eu	CD185 (CXCR5)
surface	154Sm	CD3
surface	155Gd	CD45RA
intracellular	156Gd	*IL-10
intracellular	158Gd	IL-2
intracellular	159 Tb	*IL-13
intracellular	161Dy	IL-17A
intracellular	162Dy	Foxp3
intracellular	163Dy	IL-4
surface	164Dy	CD23
intracellular	165Ho	IFNg
surface	167Er	**CD197 (CCR7)
surface	168Er	CD8a
surface	169Tm	CD25 (IL-2R)
surface	170Er	HLA-DR
intracellular	172Yb	Ki-67
surface	174Yb	CD89
surface	175Lu	CD14
surface	176Yb	CD56 (NCAM)
intracellular	191Ir	Cell-ID intercalator
intracellular	193Ir	Cell-ID intercalator
Live/dead	194Pt	Cisplatin – live/dead

**Table 3:** Immune populations assessed by biaxial gating, and the markers used for their identification; parent populations are indicated italic

Population	Markers							
Panel 1								
T cells	CD3+							
γδ Tcells	CD3+	TCRgd+						
Natural killer T cells (NKT)	CD3+	CD56+						
T double positive	CD3+	CD8+	CD4+					
T double negative	CD3+	CD8-	CD4-					
*T cytotoxic (Tc)*	*CD3+*	*CD8+*						
Tc central memory	CD3+	CD8+	CD45RA-	CCR7+				
Tc effector	CD3+	CD8+	CD45RA+	CCR7-				
Tc effector memory	CD3+	CD8+	CD45RA-	CCR7-				
Tc HLA-DR+	CD3+	CD8+	HLA-DR+					
Tc naive	CD3+	CD8+	CD45RA+	CCR7+				
*T helper (Th)*	*CD3+*	*CD4+*						
Th 2	CD3+	CD4+	CCR4+	CRTH2+/CD294				
Th central memory	CD3+	CD4+	CD45RA-	CCR7+				
Th effector	CD3+	CD4+	CD45RA+	CCR7-				
Th effector memory	CD3+	CD4+	CD45RA-	CCR7-				
Th follicular (Th FC)	CD3+	CD4+	CD4+	CD185+				
Th activated HLA-DR+	CD3+	CD4+	HLA-DR+					
Th naive	CD3+	CD4+	CD45RA+	CCR7+				
Th 1	CD3+	CD4+	CXCR3+					
Th 17	CD3+	CD4+	CXCR3-	CCR6+				
T regulatory (Tregs)	CD3+	CD4+	CD25+	CD127-				
*B cells HLA-DR+*	*CD19+*	*HLA-DR+*						
B cells double negative	CD19+	HLA-DR+	IgD-	CD27-				
B cells naive	CD19+	HLA-DR+	IgD+	CD27-				
B cells non-class-sw. mem.	CD19+	HLA-DR+	IgD+	CD27+				
B cells class-sw. mem.	CD19+	HLA-DR+	IgD-	CD27+				
Plasmablasts	CD19+	HLA-DR+	IgD-	CD27+	CD38++			
*Monocytes (Mo)*	*CD3-*	*CD19-*	*CD56-*	*CD45+*	*CD33+*	*CD14+/++*	*CD16-/+/++*	
Mo classical	CD3-	CD19-	CD56-	CD45+	CD33+	CD14++	CD16-	
Mo intermediate	CD3-	CD19-	CD56-	CD45+	CD33+	CD14++	CD16+	
Mo non-classical	CD3-	CD19-	CD56-	CD45+	CD33+	CD14+	CD16++	
Dendritic cells plasmacytoid	CD3-	CD19-	CD56-	CD45+	CD14-	HLA-DR+	CD123+	
*Dendritic cells myeloid (mDC)*	*CD3-*	*CD19-*	*CD56-*	*CD45+*	*CD14-*	*HLA-DR+*	*CD11c+*	
*Natural killer cells (NK)*	*CD3-*	*CD19-*	*CD14-*	*CD33-/+*	*CD161+*	*CD56+*		
NK precursor	CD3-	CD19-	CD14-	CD33-/+	CD161+	CD56-		
NK mature	CD3-	CD19-	CD14-	CD33-/+	CD161+	CD56+	CD45	CD56dim
NK immature	CD3-	CD19-	CD14-	CD33-/+	CD161+	CD56+	CD45	CD56bright
ILC2s	CD3-	CD19-	CD16-	CD56-	CD14-	CD161-	CD127+	CRTH2+/CD294+

## Supplementary information

**Additional file 1 Supplementaru methods**. **Supplementary Table I**. Serum levels of total and specific IgE and IgG4 and of IL-6, IL-8, TNF-α, and MCP-1 measured in serum collected at time of the study. **Supplementary Table II**. Significance levels, group median, and median marker intensity or cell abundance range for each group of participants. **Supplementary Fig. I**. CITRUS trees in which each node denotes different cell clusters. The red nodes illustrate cell populations where the median marker intensities of the respective functional marker differed statistically significantly between the three groups as determined by SAM analyses, FDR 0.05. The parent and last generation child clusters are named. **Supplementary Fig. II**. Mass cytometric analysis of stimulated cells.

## Data Availability

The datasets generated and analysed in this study are available upon request to the corresponding author.

## Abbreviations

sIgE: Allergen-specific immunoglobulin E
PBMC: Peripheral blood mononuclear cells
CyTOF: Cytometry by time of flight
RT: Room temperature
PBS: Phosphate buffered saline
FCS: Fetal calf serum
DMSO: Dimethylsulfoxid
IL: Interleukin
CCM: Cell culture medium
CITRUS: Cluster identification, characterization, and regression
SAM: Significance analysis of microarrays
PAMR: Pattern analysis of microarrays implemented in R
viSNE: Visualization of t-distributed stochastic neighbor embedding
NK cells: Natural killer cells
DC: Dendritic cells
Th cells: T helper cells, CD4+
Tc cells: Cytotoxic T cells, CD8+
