# Supplementary material for: Reduced polyfunctional T cells and increased cellular activation markers in adult allergy patients reporting adverse reactions to food
Source: BMC Immunol. 2020 Jul 22;21:43. doi: 10.1186/s12865-020-00373-w (PMC7376650; doi:10.1186/s12865-020-00373-w)
Supplement: Supplementary file 1 — Additional file 1 Supplementaru methods. Supplementary Table I. Serum levels of total and specific IgE and IgG4 and of IL-6, IL-8, TNF-α, and MCP-1 measured in serum collected at time of the study. Supplementary Table II. Significance levels, group median, and median marker intensity or cell abundance range for each group of participants. Supplementary Fig. I. CITRUS trees in which each node denotes different cell clusters. The red nodes illustrate cell populations where the median marker intensities of the respective functional marker differed statistically significantly between the three groups as determined by SAM analyses, FDR 0.05. The parent and last generation child clusters are named. Supplementary Fig. II. Mass cytometric analysis of stimulated cells. [file 12865_2020_373_MOESM1_ESM.pdf]

# Reduced polyfunctional T cells and increased cellular activation markers in adult food allergy patients

Sonnet, Friederike, MSc[1], Namork, Ellen, PhD[1], Stylianou, Eva, PhD[2], Gaare-Olstad, Ingvild, MSc[2], Huse, Kanutte, PhD[3], Andorf, Sandra, PhD[4], Mjaaland, Siri, PhD[5,6], Dirven, Hubert A.A.M., PhD[1], Nygaard, Unni C., PhD[1]

*[1]Department of Toxicology and Risk Assessment, Norwegian Institute of Public Health, Lovisenberggata 8 Oslo, Norway [2]Regional Unit for Asthma, Allergy and Hypersensitivity, Dept. of Pulmonary Diseases, Oslo University Hospital, Kirkeveien 166 Oslo, Norway [3] Department of Cancer Immunology, Oslo University Hospital, Ullernchausseen 70 Oslo, Norway [4] Sean N. Parker Center for Allergy and Asthma Research, Stanford University School of Medicine, 291 Campus Drive Stanford, CA, United States [5]Department of Infectious Disease Immunology, Norwegian Institute of Public Health, Lovisenberggata 8 Oslo, Norway, [6]K.G. Jebsen Center for Influenza Vaccine Research Oslo, Kirkeveien 166 Oslo, Norway*

Corresponding author:
Friederike Sonnet
Telephone: +49 1755703179
E-mail: F.Sonnet@lumc.nl
Address: Lomanlaan 103, 3526XD Utrecht, the Netherlands

## SUPPLEMENTARY INFORMATION

### Supplementary methods

*Blood samples and preparation of PBMC*

Blood was drawn into pyrogen-free vacuum blood collection tubes without any additives or with EDTA as anticoagulant. Blood in tubes without additives was allowed to clot for at least 1 h before centrifugation at 800 x g for 10 min at room temperature (RT), and serum was collected and stored for later analyses of total IgE, total IgG4, sIgE (stored at -20°C), and cytokines (stored at -80°C).

EDTA blood was diluted with an equal volume of physiological saline (PBS) within 2 hours after collection. PBMCs were isolated by density gradient centrifugation using Ficoll-Paque PLUS [GE Helthcare Bio-Sciences AB, Uppsala, Sweden] at 800 x g for 20 min at RT. Mononuclear cells were carefully removed and transferred to 50 ml sterile tubes. Platelets were removed by mixing gently with PBS, centrifugation at 250 x g for 10 min at RT before discarding the supernatant. After resuspension in PBS, cells from the same donor were pooled, viable cells were counted, and the washing step was repeated. After slowly adding ice-cold foetal calf serum (FCS) with 20%

dimethylsulphoxyd (DMSO, Sigma-Aldrich), cells were frozen in a CoolCell™ Freezing Container [Sigma-Aldrich] at -80 °C for 16-24 h before transferring to liquid nitrogen for storage.

*Mass Cytometry*

*Antibodies and reagents*

Forty-two pre-conjugated metal-tagged antibodies were obtained from Fluidigm [Fluidigm, CA, USA] while three purified monoclonal antibodies targeting IgG, IL-10, and IL-13 were obtained from BioLegend [BioLegend, CA, USA] and conjugated in house using Maxpar® X8 Antibody Labelling Kits [Fluidigm] according to the manufacturer's instruction. Based on titration performance, the in-house conjugated antibodies were diluted in Candor PBS-based Antibody Stabilization solution [Candor Biosciences, Germany] and stored at 4 °C. The list of antibodies used is presented in Table 2.

*Thawing of PBMC*

For each participant, cryopreserved PBMC were rapidly thawed at 37 °C in a water bath, transferred into a 50 ml Falcon™ tube [Fisher] and 9 ml of prewarmed benzonase medium (RPMI [Fisher], 10 % FCS, 1 % penicillin/streptomycin [PAA Laboratories GmbH], 25 U/ml benzonase [Sigma-Aldrich]) were added gently. Cells were pelleted (300 x g, 10 min, RT), resuspended gently in 10 ml benzonase medium, and again pelleted (300 x g, 10 min, RT). Cells were then washed in cell culture medium (CCM; RPMI, 10 % FCS, 1 % penicillin/streptomycin) (300 x g, 5 min, RT), counted, and cell numbers were adjusted to 5 x $10^6$ viable cells/ml in CCM. 3 x $10^6$ cells (600 µl) were transferred into two Corning™ Falcon™ round-bottom polystyrene tubes [Thermo Fisher Scientific Inc., Norway] and were left to rest overnight in a $CO_2$-incubator at 37 °C at 5 % $CO_2$.

*Sample preparation unstimulated cells - Panel 1*

All centrifugations were performed at 300x g for 5 min at RT unless otherwise specified.

One vial of PBMC per participant was pelleted and washed in 3 ml PBS. Cells were resuspended in 300 µl PBS with 5 µM Cell-ID™ Cisplatin [Fluidigm] for viability staining and incubated for 5 min at RT. After incubation, cells were washed in 1.5 ml Maxpar® Cell Staining Buffer [CSB; Fluidigm] and subsequently stained with 50 µl antibody cocktail, containing 0.5 µl of each of the 34 Panel 1 antibodies (Table 2) in CSB, for 30 min on ice. Cells were then washed twice with 2 ml CSB and thereafter 1 ml of 1x iridium-containing intercalator solution (4000x Cell-ID™ Intercalator-Ir [Fluidigm], diluted 1:4000 in Maxpar® Fix and Perm Buffer [Fluidigm]) was added to identify nucleated cells. Cells were left overnight at 4 °C. The next day, cells were washed twice with 2 ml PBS (800x g, 5 min, RT) and kept pelleted at RT until CyTOF data acquisition.

*Sample preparation stimulated cells - Panel 2*

All centrifugations were performed at 300x g for 5 min at RT before fixation and at 800x g after fixation.

PBMC in the second vial were stimulated in 0.6 ml CCM supplemented with 20 ng/ml PMA [phorbol 12-myristate 13-acetate; Sigma-Adrich], 1 µg/ml ionomycin [Sigma-Aldrich], and 10 µg/ml BFA [brefeldin A; Biolegend] for 4 h at 37 °C in a $CO_2$-incubator. Cells were then pelleted and washed in 3 ml PBS. Next, cells were resuspended in

300 µl PBS with 5 µM Cell-ID™ Cisplatin and incubated for 5 min at RT. After incubation, cells were washed in 1.5 ml CSB, subsequently stained with 50 µl surface antibody cocktail, containing 0.5 µl of each of the 17 Panel 2 surface antibodies (Table 2), in CSB for 30 min on ice. Cells were then washed twice with 2 ml CSB before fixated by adding 1 ml fix solution (Maxpar® Fix I Buffer (5x) [Fluidigm] diluted 1:5 in PBS] and incubated for 20 min at RT. Afterwards, cells were washed in 1 ml PBS and subsequently permeabilized by adding 1 ml ice-cold MeOH and left overnight at -20 °C. The next day, cells were rehydrated by washing first with 1 ml PBS and then with 2 ml Maxpar® Perm-S Buffer [Fluidigm] prior to staining with 50 µl intracellular antibody cocktail containing 0.5 µl of each of the 11 Panel 2 intracellular antibodies (Table 2) in Maxpar® Perm-S Buffer, for 30 min at RT. Cells were washed twice with 2 ml CSB before 1 ml of 1x iridium-containing intercalator solution was added and incubated 20 min at RT. Subsequently, cells were washed twice with 2 ml PBS and kept pelleted at RT until CyTOF data acquisition.

*Data analyses*

Bead-normalized files were analyzed following two complementary approaches in Cytobank [Cytobank Inc.]. Living intact single cells were selected before downstream analysis, based on DNA content and event length as $Ir191^+$ $Ir193^+$ $CD45^+$ $Cisplatin^-$ events from which EQ beads and double positive $CD3^+CD14^+$ and $CD3^+CD19^+$ events were excluded.

In the first, supervised approach, cell subsets were identified using a manual gating strategy to immunophenotype cells with canonical cell surface markers and to assign functional attributes (e.g., activation state or cytokine production). Cell subpopulations and the markers used for their identification are presented in Table 3.

The second approach applied the CITRUS algorithm (cluster identification, characterization, and regression (Cytobank)). CITRUS first performs an unsupervised hierarchical clustering to identify clusters of cellular populations within the overall dataset, displaying a hierarchy of phenotypically related cell clusters. Next, biologically relevant features (i.e. relative cell abundance or median marker expression) of these clustered populations are calculated per sample, and correlative (SAM) or predictive (PAMR) methods are used to identify the cell clusters (populations) that differ significantly between the patient groups with regard to cell abundance or median marker intensities (15). CITRUS analysis was performed on living intact single cells with the default configuration (arcsinh cofactor = 5, normalize = “global”, down-sampling events = 3000, downsampling exclusion percentile = 0.01, target number of clusters = 250, maximum number of clustered events = 50000) and a 2 % cutoff for cluster size. For unstimulated cells (panel 1), clustering was performed on i) all 33 markers and ii) 25 phenotyping markers excluding the functional/activation markers (CD23, CD25, CD28, CD69, CD123, CD134, CD371, and HLA-DR). For stimulated cells (panel 2), clustering was performed using all 28 markers except CCR7, since only half of the samples contained CCR7 due to an error. All CITRUS analyses were repeated twice to assure reproducibility.

The multiple testing permutation procedure (SAM), with a false discovery rate of 0.05 were used to identify cell populations differing significantly between groups. For these populations, pairwise comparison between the cell abundance and mean marker intensities in the three groups (healthy donors, IgEpos, and IgEneg food allergic subjects) were performed by exporting the data and using the Kruskal-Wallis test and Dunn’s multiple comparison

post-hoc test in GraphPad Prism 5 [GraphPad Software, Inc.]. P values of less or equal than 0.05 were considered statistically significant and denoted as follows: * $P \leq 0.05$, ** $P \leq 0.01$, *** $P \leq 0.001$, and **** $P \leq 0.0001$. Results were visualized in box plots prepared by CITRUS.
The PAMR results were used for verification purposes.
Phenotype characterization of the significant cell clusters was performed based on histograms provided by the software for each marker, where the red histograms show the expression of the marker in the cluster and blue the expression of the marker in the background clusters. Presented box plots, showing cell abundance or median marker intensities for each individual as well as group median and quartiles, were also provided by the Cytobank software.

For data visualization, viSNE (visualization of t-Distributed Stochastic Neighbour Embedding) analysis for stimulated cells (panel 2) was performed for all participants with the default configuration (seed: (random), # iterations: 1000, perplexity: 30, theta: 0,5). All 28 markers (excluding CCR7) were used for clustering and 100.000 events were sampled equally. The major immune cell populations were identified based on marker expression denoted in Table 3.

## Supplementary tables

Supplementary Table I. Serum levels of total and specific IgE and IgG4 and of IL-6, IL-8, TNF-α, and MCP-1 measured in serum collected at time of the study.
Bold denotes positive (> 0.35 kU/L) sIgE levels in serum.

| | **Tot-IgG4** | **Tot-IgE** | **Tot-IgG4/ Tot-IgE ratio** | **Specific IgE to the allergens in the standard panel** (kU/l) | | | | | | | | | | | | | | **Additional positive food allergens[2]** | **Additional positive airway allergens[3]** | **IL-6** | **IL-8** | **TNFa** | **MCP-1** |
|---|---|---|---|---|---|---|---|---|---|---|---|---|---|---|---|---|---|---|---|---|---|---|---|
| | (ng/ml) | (ng/ml) | | **Milk** | **Egg** | **Wheat** | **Pea** | **Soy** | **Peanut** | **Fenugreek** | **Hazelnut** | **Celery** | **Cod** | **Salmon** | **Shrimp** | **Birch[4]** | **Timothy[4]** | (kU/L) | (kU/L) | **(pg/ml)** | **(pg/ml)** | **(pg/ml)** | **(pg/ml)** |
| ***IgEneg donors*** | | | | | | | | | | | | | | | | | | | | | | | |
| **N1** | 118817 | 3.36 | 35404 | 0 | 0 | 0 | 0 | 0 | 0 | 0 | 0 | 0 | 0 | 0 | 0 | neg | neg | | | 5.3 | 5.03 | 5.46 | 307.07 |
| **N2** | 3373311 | 22.15 | 152280 | 0 | 0 | 0 | 0 | 0 | 0 | 0 | 0 | 0 | 0 | 0 | 0 | neg | neg | | | 0 | 2.58 | 2.05 | 303.46 |
| **N3** | 307710 | 121.58 | 2531 | 0 | 0 | 0 | 0 | 0 | 0 | 0 | 0 | 0 | 0 | 0 | 0 | neg | neg | | | 0.46 | 0.37 | 3.74 | 226.57 |
| **N4** | 673516 | 9.89 | 68114 | 0 | 0 | 0 | 0 | 0 | 0 | 0 | 0 | 0 | 0 | 0 | 0 | neg | neg | | | 4.11 | 2.66 | 2.8 | 271.13 |
| **N5** | 4623546 | 7.08 | 653043 | 0 | 0 | 0 | 0 | 0 | 0 | 0 | 0 | 0 | 0 | 0 | 0 | neg | neg | | | 0 | 12.21 | 1.09 | 337.91 |
| **N6** | 101705 | 68.88 | 1477 | 0 | 0 | 0 | 0 | 0 | 0 | 0 | 0 | 0 | 0 | 0 | 0 | neg | neg | | | 1 | 1.65 | 7.9 | 94.99 |
| **N7** | 1363093 | 7.08 | 192473 | 0 | 0 | 0 | 0 | 0 | 0 | 0 | 0 | 0 | 0 | 0 | 0 | neg | neg | | | 0.2 | 1.69 | 4.02 | 364.48 |
| **N8** | 5140192 | 5002.00 | 1028 | 0 | 0 | 0 | 0 | 0 | 0 | 0 | 0 | 0 | 0 | 0 | 0 | neg | neg | | | 0.41 | 2.27 | 2.79 | 48.99 |
| **N9** | 574434 | 72.08 | 7969 | 0 | 0 | 0 | 0 | 0 | 0 | 0 | 0 | 0 | 0 | 0 | 0 | neg | neg | | | 0.81 | 2.9 | 3.48 | 480.61 |
| **Group median** | **673516** | **22.152** | **35404** | **0.02** | **0** | **0.03** | **0.01** | **0.01** | **0.02** | **0.05** | **0.01** | **0.01** | **0.01** | **0.01** | **0.02** | | | | | **5.3** | **5.03** | **5.46** | **307.07** |
| ***IgEpos donors*** | | | | | | | | | | | | | | | | | | | | | | | |
| **P1** | 329644 | 162.83 | 2024 | 0.04 | 0.1 | 0.03 | 0.01 | 0.02 | 0.04 | 0.06 | **0.58** | 0.03 | 0.01 | 0.06 | 0.01 | **1.1** | **1.2** | | **cat 5.2, dog 0.91** | 0 | 2.71 | 0.87 | 217.12 |
| **P2** | 1794944 | 224.84 | 7983 | 0.02 | 0 | 0.17 | 0.02 | 0.03 | 0.11 | 0.08 | **0.41** | 0.07 | 0.01 | 0.02 | 0.05 | **3.8** | **5.9** | | **cat 2.8, dog 10.0, horse 1.5, mugw. 1.38** | 0.09 | 3.23 | 2.59 | 135.56 |
| **P3** | 2064518 | 24.88 | 82989 | 0.06 | 0 | **0.71** | **0.75** | 0.29 | **1.27** | 0.11 | **1.74** | 0.04 | 0.01 | 0.02 | 0.01 | **2.23** | **0.74** | | **mugw. 0.87** | 0.31 | 1.58 | 4.76 | 210.44 |
| **P4** | 1561817 | 210.32 | 7426 | 0.05 | 0.1 | 0.04 | 0.02 | 0.01 | 0.01 | 0.04 | 0.01 | 0.02 | 0.01 | 0.01 | 0.18 | 0 | **9** | | **mugw. 0.36, dog 2.5** | 0 | 0.01 | 2.09 | 169.74 |
| **P5** | 841217 | 125.01 | 6729 | 0.02 | 0 | 0.03 | **2.85** | **2.31** | **0.47** | 0.19 | **6.42** | 0.01 | 0.01 | 0.02 | 0.01 | **4.8** | 0 | | **cat 0.36** | 1.09 | 2.95 | 2.87 | 139.28 |
| **P6** | 2107904 | 6.85 | 307768 | 0.01 | 0 | 0.02 | 0.01 | 0 | 0.02 | 0.05 | 0.01 | 0.01 | 0.01 | 0.01 | 0.01 | 0 | 0 | **chicken 2.76** | | 1.56 | 1.06 | 2.04 | 162.42 |
| **P7** | 590877 | 1257.77 | 470 | 0.07 | 0.2 | 0.23 | 0.03 | 0.04 | 0.11 | 0.1 | **3.72** | 0.09 | 0.03 | 0.03 | 0.09 | **16** | **7.59** | | **cat 2.0, dog 1.9, horse 0.78, mugw. 1.6** | 0 | 1.63 | 2.82 | 206.72 |
| **P8** | 2133637 | 934.63 | 2283 | 0.05 | 0.1 | 0.26 | 0.05 | 0.1 | **0.9** | **0.38** | **3.85** | **2.04** | 0.02 | 0.01 | 0.02 | **17.2** | **21.9** | **chili 1.2, parsley 1.6** | **cat 39, dog 6.1, horse 1.0, mugw. 21.8** | 2.9 | 1.62 | 3.27 | 238.65 |
| **P9** | 1170620 | 3678.71 | 318 | 0.09 | 0.1 | 0.05 | 0.03 | 0.02 | 0.03 | 0.06 | 0.03 | 0.03 | 0.02 | 0.03 | **1.33** | 0 | 0 | **lupine 4.2** | **HDM 4.17** | 0 | 1.09 | 1.76 | 122.96 |
| **P10** | 239241 | 1331.59 | 180 | 0.03 | 0 | 0.05 | 0.01 | 0.04 | **0.5** | 0.11 | **6.35** | **0.38** | 0.01 | 0.02 | 0.03 | **20** | **2.4** | | **cat 0.45, dog 1.7, horse 2.6** | 3.64 | 2.1 | 4.95 | 137.03 |
| **P11** | 6115659 | 5002.00 | 1223 | 0.11 | 0.1 | **11.5** | **42.1** | **12.5** | **4.83** | **21.8** | **8.06** | 0.13 | 0.03 | 0.04 | 0.29 | **14** | **24** | | **cat 11.0, dog 1.1, horse 0.8, HDM 9.0** | 0 | 0.86 | 1.48 | 125.82 |
| **Group median** | **1561817** | **224.836** | **2283** | **0.05** | **0.1** | **0.05** | **0.03** | **0.04** | **0.11** | **0.1** | **1.74** | **0.04** | **0.01** | **0.02** | **0.03** | **3.8** | **2.4** | | | **0.09** | **1.62** | **2.59** | **162.42** |
| ***Healthy donors*** | | | | | | | | | | | | | | | | | | | | | | | |

| | | | | | | | | | | | | | | | | | | | | | | | |
|---|---|---|---|---|---|---|---|---|---|---|---|---|---|---|---|---|---|---|---|---|---|---|---|
| | n.a | n.a. | n.a. | 0.02 | 0 | 0.03 | 0.01 | 0.01 | 0.03 | 0.06 | 0.01 | n.a. | 0.01 | 0.02 | 0.01 | neg | neg | | | 0 | 3.61 | 4.64 | 233.2 |
| | 18065 | 5.03 | 3589 | 0.01 | 0 | 0.02 | 0.01 | 0 | 0.02 | 0.06 | 0.01 | 0.01 | 0 | 0.01 | 0.01 | neg | neg | | | 0 | 2.15 | 1.4 | 125.77 |
| | 5916195 | 107.67 | 54946 | 0.02 | 0 | 0.02 | 0.01 | 0.01 | 0.02 | 0.08 | 0.01 | 0.01 | 0 | 0.01 | 0.01 | neg | neg | | | 0 | 2.1 | 1.32 | 190.6 |
| | 3249888 | 236.14 | 13763 | 0.02 | 0 | 0.02 | 0.01 | 0 | 0.02 | 0.05 | 0.01 | 0.01 | 0.01 | 0.01 | 0.02 | neg | neg | | | 4.11 | 3.31 | 3.18 | 340.86 |
| | 1860022 | 122.00 | 15246 | 0.03 | 0 | 0.03 | 0.01 | 0.01 | 0.02 | 0.06 | 0.01 | 0.01 | 0.01 | 0.02 | 0.02 | neg | neg | | | 0 | 2.16 | 2.53 | 223.59 |
| | 922757 | 138.69 | 6653 | 0.03 | 0 | 0.02 | 0.01 | 0.01 | 0.02 | 0.07 | 0.02 | 0.01 | 0.01 | 0.03 | 0.02 | neg | neg | | | 0 | 1.44 | 1.58 | 164.36 |
| | 650300 | 154.77 | 4202 | 0.13 | 0 | 0.03 | 0.01 | 0.01 | 0.02 | 0.05 | 0 | 0.01 | 0 | 0.02 | 0.01 | neg | neg | | | 0 | 2.55 | 2.46 | 149.32 |
| | 1157307 | 24.65 | 46953 | 0.02 | 0 | 0.02 | 0.01 | 0.01 | 0.02 | 0.05 | 0 | 0 | 0.01 | 0.02 | 0.01 | neg | neg | | | 0 | 1.61 | 1.45 | 116.12 |
| **Group median** | **1157307** | **122.003** | **13763** | **0.02** | **0** | **0.02** | **0.01** | **0.01** | **0.02** | **0.06** | **0.01** | **0.01** | **0.01** | **0.02** | **0.01** | | | | | **0** | **2.155** | **2.02** | **177.48** |

Supplementary Table II. Significance levels, group median, and median marker intensity or cell abundance range for each group of participants.

ctr/+ denotes the comparison between the control and the IgEpos allergy group, ctr/- denotes the comparison between the control and the IgEneg group, +/- denotes the comparison between the IgEpos and IgEneg allergy groups, respectively. * denotes P ≤ 0.05, ** denotes P ≤ 0.01, *** denotes P ≤ 0.001, **** denotes P ≤ 0.0001

A) Median marker intensities in the unstimulated cells

B) Cell abundance in the stimulated cells

C) Median marker intensities in the stimulated cells

A)

| Cluster | | Marker | Significance | | | Median marker intensity (median) | | | Range (min. to max.) | | | | | |
|---|---|---|---|---|---|---|---|---|---|---|---|---|---|---|
| | | | | | | | | | ctr | | IgEpos | | IgEneg | |
| Parent | Child | | ctr/+ | ctr/- | +/- | ctr | IgEpos | IgEneg | min | max | min | max | min | max |
| Mo#1 | | CD371 | ** | *** | | 2.87 | 3.25 | 3.41 | 2.06 | 2.99 | 2.97 | 3.59 | 3.03 | 3.73 |
| | Mo#1a | CD371 | ** | ** | | 3.04 | 3.33 | 3.29 | 2.71 | 3.08 | 3.10 | 3.64 | 3.04 | 3.76 |
| | Mo#1b | CD371 | ** | *** | | 2.81 | 3.26 | 3.25 | 2.25 | 2.93 | 3.02 | 3.47 | 2.96 | 3.68 |
| Mo#1 | | CD69 | | ** | | 1.53 | 2.06 | 2.04 | 1.03 | 1.91 | 1.38 | 2.62 | 1.75 | 3.17 |
| | Mo#1a | CD69 | * | * | | 1.50 | 2.06 | 2.20 | 1.01 | 1.82 | 1.36 | 2.47 | 1.12 | 3.12 |
| | Mo#1b | CD69 | * | ** | | 1.53 | 2.19 | 2.15 | 0.93 | 1.78 | 1.56 | 2.57 | 1.88 | 3.32 |
| Mo#1 | | CD23 | | ** | | 0.21 | 0.03 | 0.00 | 0.12 | 0.34 | 0.00 | 0.35 | 0.00 | 0.10 |
| | Mo#1a | CD23 | ** | *** | | 0.11 | 0.00 | 0.00 | 0.07 | 0.25 | 0.00 | 0.13 | 0.00 | 0.04 |
| | Mo#1b | CD23 | | ** | | 0.32 | 0.16 | 0.08 | 0.20 | 0.50 | 0.00 | 0.40 | 0.00 | 0.21 |
| Th#1 | | CD25 | *** | * | | 1.24 | 1.85 | 1.75 | 1.15 | 1.46 | 1.43 | 2.09 | 1.41 | 1.99 |
| | Th#1a | CD25 | * | | | 0.83 | 1.11 | 1.02 | 0.27 | 1.06 | 0.92 | 1.77 | 0.46 | 1.47 |
| | Th#1b | CD25 | *** | * | | 1.53 | 2.28 | 1.98 | 1.34 | 1.53 | 1.66 | 2.28 | 1.51 | 1.98 |
| | Th#1c | CD25 | ** | | | 1.09 | 1.53 | 1.39 | 0.99 | 1.46 | 1.14 | 1.83 | 0.98 | 2.17 |
| | Th#1d | CD25 | * | * | | 1.13 | 1.44 | 1.42 | 0.54 | 1.35 | 0.91 | 1.60 | 1.17 | 1.92 |
| Th#2 | | CD23 | **** | *** | | 0.05 | 0.00 | 0.00 | 0.01 | 0.13 | 0.00 | 0.01 | 0.00 | 0.00 |
| | Th#2a | CD23 | *** | **** | | 0.08 | 0.00 | 0.00 | 0.02 | 0.13 | 0.00 | 0.10 | 0.00 | 0.00 |
| Th#3 | | CD23 | **** | **** | | 0.05 | 0.00 | 0.00 | 0.01 | 0.09 | 0.00 | 0.00 | 0.00 | 0.00 |
| | Th#3a | CD23 | **** | **** | | 0.05 | 0.00 | 0.00 | 0.01 | 0.09 | 0.00 | 0.00 | 0.00 | 0.00 |
| | Th#3b | CD23 | *** | ** | | 0.07 | 0.00 | 0.00 | 0.00 | 0.10 | 0.00 | 0.00 | 0.00 | 0.04 |
| Th#4 | | CD25 | ** | | * | 1.77 | 2.31 | 1.64 | 1.09 | 2.06 | 1.46 | 2.74 | 0.63 | 2.29 |
| Th#4 | | CD28 | * | | | 3.51 | 3.81 | 3.55 | 3.07 | 3.71 | 3.39 | 4.16 | 2.69 | 4.33 |
| Tc#1 | | CD28 | ** | * | | 3.07 | 3.42 | 3.36 | 1.80 | 3.23 | 2.99 | 3.68 | 2.70 | 3.81 |
| | Tc#1a | CD28 | ** | * | | 3.21 | 3.59 | 3.56 | 2.69 | 3.38 | 2.95 | 3.94 | 3.16 | 3.93 |
| Tc#2 | | HLA-DR | * | | | 0.72 | 1.26 | 0.96 | 0.35 | 1.14 | 0.60 | 2.16 | 0.52 | 2.02 |
| | Tc#2a | HLA-DR | * | | | 0.69 | 1.34 | 0.97 | 0.34 | 1.05 | 0.59 | 1.97 | 0.00 | 2.07 |
| B#1 | | HLA-DR | ** | | | 5.28 | 5.98 | 5.65 | 4.93 | 5.49 | 5.25 | 6.39 | 5.04 | 6.25 |
| | B#1a | HLA-DR | ** | | | 5.32 | 5.99 | 5.61 | 4.97 | 5.52 | 5.29 | 6.41 | 5.04 | 6.16 |
| | B#1b | HLA-DR | * | | | 5.24 | 5.95 | 5.61 | 4.87 | 5.52 | 5.20 | 6.39 | 5.05 | 6.32 |

B)

| Cluster | | Significance | | | Abundance (median) | | | ctr | | IgEpos | | IgEneg | |
|---|---|---|---|---|---|---|---|---|---|---|---|---|---|
| Parent | Child | ctr/+ | ctr/- | +/- | ctr | IgEpos | IgEneg | min | max | min | max | min | max |
| Th | | | * | | 0.0616 | 0.0188 | 0.0040 | 0.0006 | 0.1328 | 0.0004 | 0.0750 | 0 | 0.0454 |
| | Th1 | | * | | 0.0436 | 0.0130 | 0.0014 | 0.0004 | 0.1000 | 0.0002 | 0.0620 | 0 | 0.027 |
| Tc | | | ** | | 0.0942 | 0.0112 | 0.0060 | 0.0040 | 0.2172 | 0.0032 | 0.1384 | 0 | 0.0542 |
| | Tc1 | | * | | 0.0398 | 0.0058 | 0.0014 | 0.0010 | 0.1240 | 0.0022 | 0.0548 | 0 | 0.028 |

C)

| | | Significance | | | Median marker intensity (median) | | | ctr | | IgEpos | | IgEneg | |
|---|---|---|---|---|---|---|---|---|---|---|---|---|---|
| Cluster | Marker | ctr/+ | ctr/- | +/- | ctr | IgEpos | IgEneg | min | max | min | max | min | max |
| sTc#1 | IFN-g | | ** | | 3.799 | 3.140 | 2.007 | 0.978 | 4.293 | 1.686 | 4.030 | 0 | 3.712 |
| sTc#1 | TNF-a | | | | 2.855 | 2.302 | 1.517 | 2.275 | 3.196 | 1.863 | 3.509 | 0 | 3.582 |
| sNK#1 | IFN-g | | * | * | 2.650 | 2.661 | 1.413 | 0 | 2.894 | 1.77 | 3.137 | 0 | 2.585 |
| sNK#1 | TNF-a | | | * | 1.228 | 1.703 | 0.313 | 0 | 2.488 | 0 | 2.437 | 0 | 1.317 |
| sTh#1 | IL-17A | * | | | 0 | 0.271 | 0.066 | 0 | 0.342 | 0 | 0.934 | 0 | 0.482 |
| sTh#1 | TNF-a | | | | 0.026 | 0 | 0 | 0 | 0.187 | 0 | 0.078 | 0 | 0.013 |
| aTh#2 | TNF-a | | * | | 0.624 | 0.023 | 0 | 0 | 1.79 | 0 | 1.074 | 0 | 0.839 |
| sTh#3 | TNF-a | | * | | 0.252 | 0 | 0 | 0 | 0.844 | 0 | 0.368 | 0 | 0.357 |
| sB#1 | IL-2 | | * | | 0.526 | 0.317 | 0.279 | 0.119 | 0.840 | 0 | 0.776 | 0.114 | 0.497 |

## Supplementary figures

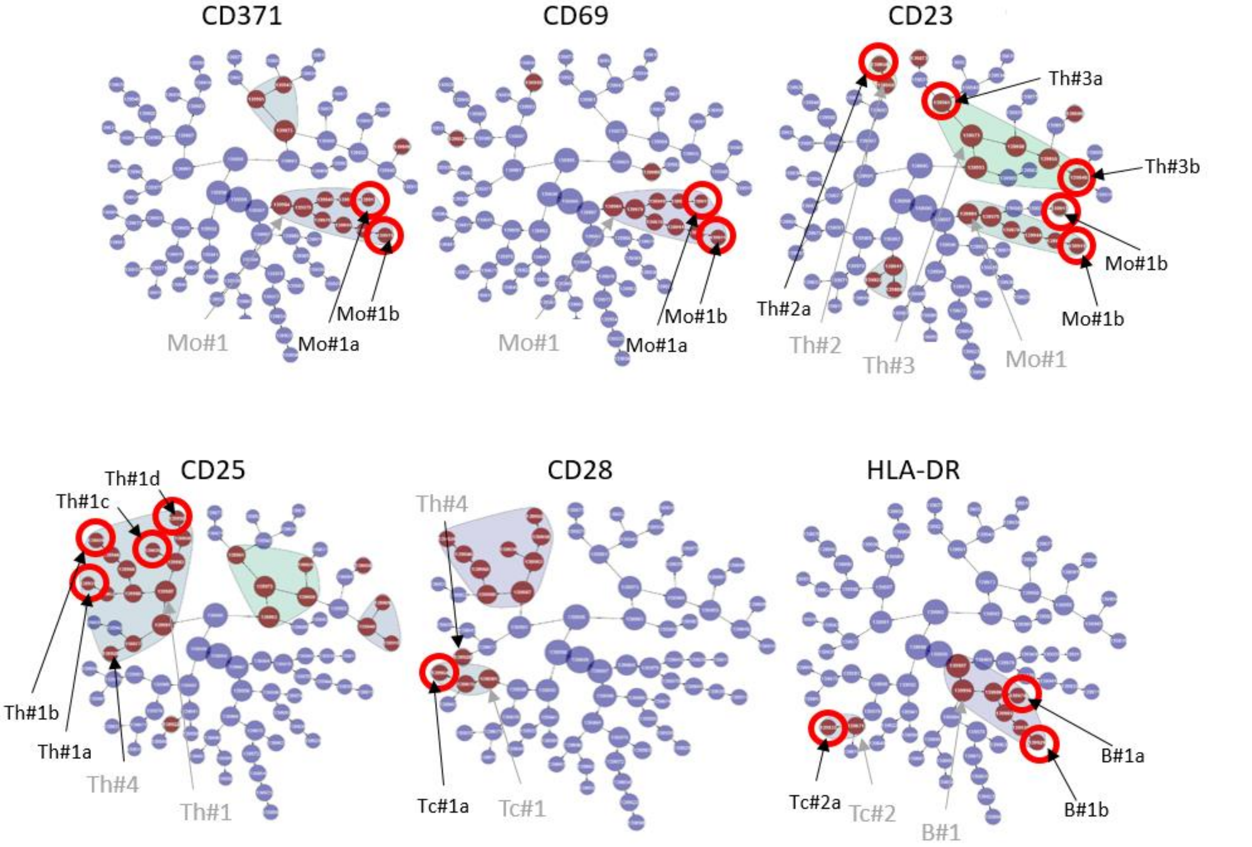


Supplementary Figure I: CITRUS trees in which each node denotes different cell clusters. The red nodes illustrate cell populations where the median marker intensities of the respective functional marker differed statistically significantly between the three groups as determined by SAM analyses, FDR 0.05. The parent and last generation child clusters are named.

A)

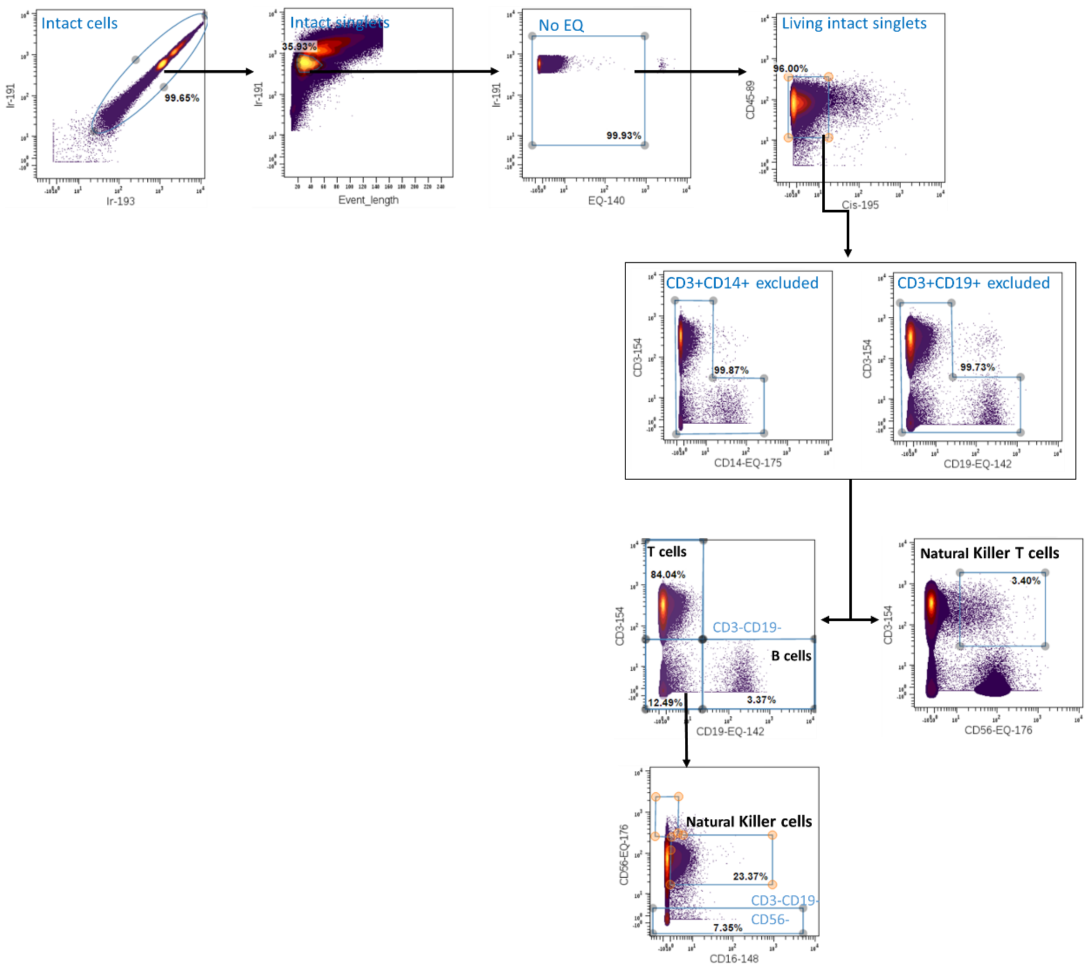
Intact cells
99.65%
Ir-191
Ir-193
Intact singlets
35.93%
Event_length
No EQ
99.93%
EQ-140
Living intact singlets
96.00%
CD45-89
Cis-195
CD3+CD14+ excluded
99.87%
CD3-154
CD14-EQ-175
CD3+CD19+ excluded
99.73%
CD19-EQ-142
T cells
84.04%
CD3-CD19-
B cells
12.49%
3.37%
Natural Killer T cells
3.40%
CD56-EQ-176
Natural Killer cells
23.37%
CD3-CD19-
CD56-
7.35%
CD16-148

B)

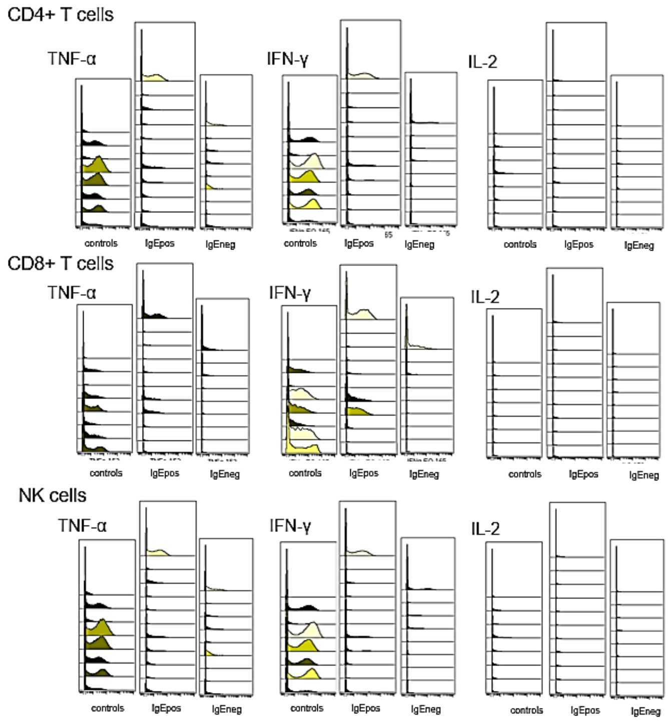
CD4+ T cells
TNF-α
IFN-γ
IL-2
controls
IgEpos
IgEneg
CD8+ T cells
NK cells

**C**)

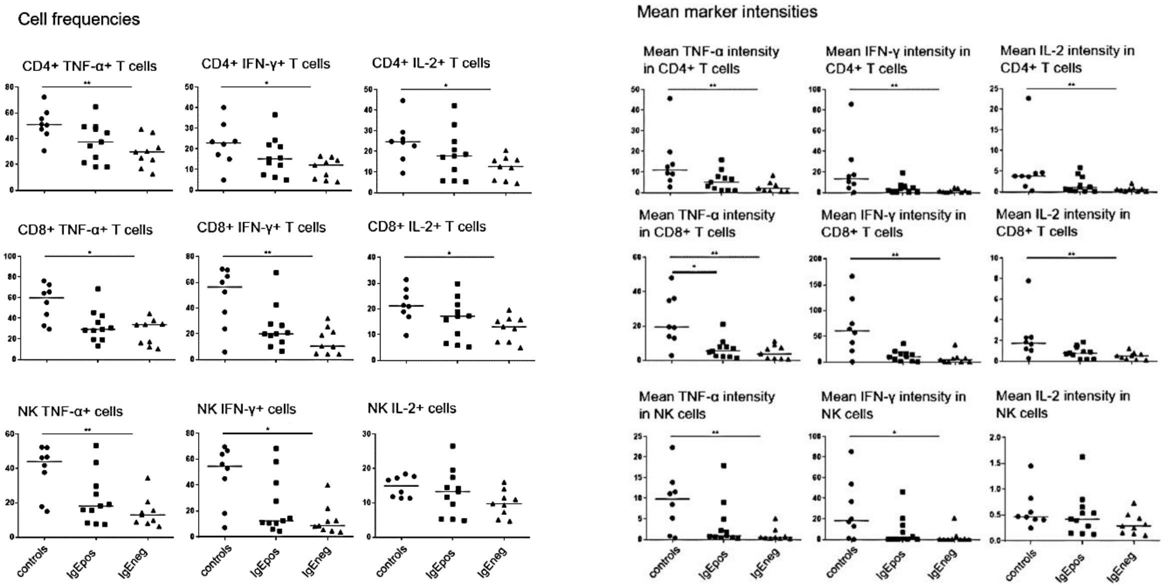


Supplementary Figure II. Mass cytometric analysis of stimulated cells.

A) Representative gating strategy of T cells, B cells, and NK cells.

B) Histograms showing the expression of TNF-α, IFN-γ, and IL-2 in T cells, B cells, and NK cells in the controls, IgEpos food allergy group, and IgEneg food allergy group.

C) Scatter plots of frequencies of TNF-α, IFN-γ, and IL-2 expressing $CD4^+$ T cells, $CD8^+$ T cells, and NK cells and mean TNF-α, IFN-γ, and IL-2 marker intensities in $CD4^+$ T cells, $CD8^+$ T cells, and NK cells in the three groups of participants: controls, IgEpos food allergy patients, and IgEneg food allergy patients. Each dot represents a participant while the lines represent the group median. * denotes $P \leq 0.05$ and ** denotes $P \leq 0.01$
